# Home mechanical ventilation: quality of life patterns after six months of treatment

**DOI:** 10.1186/s12890-020-01262-z

**Published:** 2020-08-17

**Authors:** Luca Valko, Szabolcs Baglyas, V. Anna Gyarmathy, Janos Gal, Andras Lorx

**Affiliations:** 1 Department of Anesthesiology and Intensive Therapy, Semmelweis University, Ulloi ut 78/B, Budapest, 1082 Hungary; 2 EpiConsult, Dover, DE USA; 3 Johns Hopkins Bloomberg School of Public Health, Károly Racz School of PhD Studies, Semmelweis University, 8 the Green, STE A, Dover, DE 19904 USA

**Keywords:** Home mechanical ventilation, Quality of life change, Chronic respiratory failure, Long term ventilation, Domiciliary ventilation

## Abstract

**Background:**

It has been shown that home mechanical ventilation improves quality of life, but it has not been widely studied which particular patient groups benefit the most from starting this type of therapy. The purpose of this prospective observational study was to evaluate quality of life change patterns 6 months after initiation of home mechanical ventilation in patients suffering from chronic respiratory failure using patient reported outcomes.

**Methods:**

We enrolled 74 chronic respiratory failure patients starting invasive or noninvasive home mechanical ventilation through the Semmelweis University Home Mechanical Ventilation Program. Quality of life was evaluated at baseline and at 6 months after initiation of home mechanical ventilation using the Severe Respiratory Insufficiency Questionnaire.

**Results:**

Overall quality of life showed 10.5% improvement 6 months after initiation of home mechanical ventilation (*p* < 0.001). The greatest improvement was observed in Respiratory complaint (20.4%, *p* = 0.015), Sleep and attendant symptoms (19.3%, *p* < 0.001), and Anxiety related subscales (14.4%, *p* < 0.001). Interface (invasive versus noninvasive ventilation) was not associated with improvement in quality of life (*p* = 0.660). Severely impaired patients showed the greatest improvement (CC = -0.328, *p* < 0.001). Initial diagnosis contributed to the observed change (*p* = 0.025), with chronic obstructive pulmonary disease and obesity hypoventilation syndrome patients showing the greatest improvement, while amyotrophic lateral sclerosis patients showed no improvement in quality of life. We found that patients who were started on long term ventilation in an acute setting, required oxygen supplementation and had low baseline quality of life, showed the most improvement during the six-month study period.

**Conclusions:**

Our study highlights the profound effect of home mechanical ventilation on quality of life in chronic respiratory failure patients that is indifferent of ventilation interface but is dependent on initial diagnosis and some baseline characteristics, like acute initiation, oxygen supplementation need and baseline quality of life.

**Trial registration:**

This study was approved by and registered at the ethics committee of Semmelweis University (SE TUKEB 251/2017; 20th of December, 2017).

## Background

Chronic respiratory failure affects many people, causing hypoxia, hypercapnia, secondary symptoms, diminished health related quality of life (HRQL) and adverse outcomes. Long term mechanical ventilation, which can be supplied through a noninvasive (mask) or invasive interface (tracheostomy) if noninvasive ventilation is contraindicated or not feasible (e.g. in the case of bulbar symptoms), improves outcomes in many different types of chronic respiratory failure [1–3]. Home mechanical ventilation (HMV) reduces costs, infection rates, optimizes medical care utilization and perhaps most importantly for the patients, improves HRQL [4]. Hence home mechanical ventilation has been an increasing practice worldwide [5, 6]. As guidelines do not include specific ventilation goals and settings, practice varies greatly in different regions [7]. Because of this, HRQL measurements are important tools for quality control, optimization of therapy and even outcome prediction [8].

HRQL is usually measured through questionnaires and as general health quality surveys might be inaccurate in specific diseases groups, there have been attempts to establish a more focused questionnaire useful in chronic respiratory failure patients [9–11]. Perhaps the most efficient questionnaire for this purpose designed up until now is the SRI Questionnaire, which is a multimodal tool with high psychometric properties, specifically designed to evaluate HRQL in patients battling chronic respiratory conditions [12]. The SRI Questionnaire has been validated in several different conditions and has been proven to be a superior HRQL evaluation tool for patients receiving home mechanical ventilation [9, 13]. The questionnaire has been used to evaluate HRQL in both invasively and noninvasively ventilated patients and has been validated in several different languages, including Hungarian [13–21].

The effect of starting home mechanical ventilation on patients’ HRQL has been studied before, but these studies either used a general HRQL survey [22] or focused only on a well-defined, selected population often excluding patients with tracheostomas, or patients that were recruited after inability to wean or after an acute worsening of their chronic condition [13]. As a considerable proportion of home mechanical ventilation patients are started on long term therapy after acute exacerbation or failure to wean from ventilatory support, these studies might not reflect real-life populations, as more severe patients are underrepresented (see Table 1). Studies have also shown that HRQL differs greatly in chronic respiratory failure patients with different diseases (see Table 1) and we can expect that the change in HRQL induced by home mechanical ventilation also varies [7, 10, 13, 14]. The factors that can possibly affect change in HRQL are baseline characteristics, initial diagnosis, initial HRQL, type of interface used for ventilation, duration of ventilation and lung function test parameters. Studies performed on specific patient groups might have different follow-up plans, and their HRQL changes might not be comparable.

**Table 1:** Studies utilizing SRI to report detailed HRQL for HMV patients

Author	Study aim		Patients	Main HRQL (SRI SS) finding for HMV patients
Windisch et al. [13]	Studies reporting change in HRQL	HRQL improvement during HMV	85 stable NIV patients	49 ± 15 (baseline) 61 ± 15 (1 month) 61 ± 16 (1 year)
Struik et al. [23]		NIV vs. standard treatment	108 COPD patients after acute exacerbation	47.9 ± 15.1 (baseline) 55.0 ± 15.4 (12 months)
Murphy et al. [24]		O2 therapy vs NIV + O2 therapy	64 hypercapnic patients after acute exacerbation	50.6 (6 weeks)
Howard et al. [25]		CPAP vs Bi-level PAP	57 OHS patients (outpatient or hospital referral)	50.63 ± 3.65 (baseline) 63.5 ± 3.74 (3 months)
Storre et al. [26]		AVAPS in OHS	10 OHS patients starting NIV	63 ± 15 (baseline) 78 ± 14 (6 weeks BPV-S/T) 76 ± 16 (6 weeks BPV-S/T-AVAPS)
Murphy [27]	Studies reporting HRQL for a specific HMV patient group	High intensity vs high pressure NIV	7 COPD with established HMV	57 ± 11 vs 69 ± 16
Storre et al. [28]		High intensity vs target volume NIV	10 COPD patients with established HMV	59.3 ± 14.8 vs 62.4 ± 18.9
Arellano-Maric et al. [29]		NIV vs CPAP	42 OHS patients with established NIV	61.2 ± 16 vs 65.3 ± 14
Windisch [30]		SRI validation in COPD	162 COPD patients with established NIV	52 ± 17
Walterspracher [31]		SRI for LOT COPD patients	42 COPD patients with established NIV	53.2 ± 18.6
Oga et al. [11]		HRQL tool comparisons	56 COPD/TB patients with established NIV	56.0 ± 15.3
Chen et al. [18]		Chinese SRI validation	149 stable NIV patients	52.93 ± 15.11
Budweiser [8]	Studies reporting HRQL for mixed HMV patient group	Prognostic value of HRQL	231 stable IV and IV patients	61.2 ± 17.7 (all patients) 52.2 ± 15.6 (COPD) 66.2 ± 17.2 (RCWD) 55.3 ± 9.2 (NMD) 71.3 ± 15.7 (OHS/OL)
Gosh et al. [16]		English SRI validation	152 stable NIV and IV patients	55.9 ± 18.9 (all patients) 43.1 ± 17.3 (COPD) 61.9 ± 16.1 (RCWD) 58.8 ± 20.3 (NMD) 53.4 ± 18.8 (OHS) 53.5 ± 19.7 (miscellaneous)
Oga et al. [19]		Japanese SRI validation	56 stable NIV patients	56.0 ± 15.3 (all patients) 56.6 ± 14.7 (COPD) 55.5 ± 16.4 (Tb)
Ribeiro et al. [20]		Portuguese SRI validation	93 stable NIV and IV patients	56.6 ± 15.7 (all patients) 57.0 ± 16.5 (COPD) 55.6 ± 15.1 (OHS) 62.0 ± 12.6 (RCWD) 50.2 ± 16.2 (COPD+OSA) 59.4 ± 19.2 (NMD) 46.0 ± 13.3 (miscellaneous)
Markussen et al. [17]		Norwegian SRI validation	127 stable NIV and IV patients	55.8 ± 18.4 (all patients) 61.0 ± 14.7 (NMD) 43.2 ± 19.0 (COPD) 58.4 ± 18.3 (OHS) 55.8 ± 18.4 (RCWD)
Valko et al. [21]		Hungarian SRI validation	104 stable NIV and IV patients	66.8 ± 15.1 (NIV) 58.2 ± 13.6 (IV)
Huttmann et al. [32]		HRQL after unsuccessful weaning	25 IV patients	49 ± 16 (NMD) 47 ± 20 (COPD)
Huttmann et al. [14]		HRQL of invasively ventilated HMV patients	32 IV patients	53 ± 16 (all patients) 58 ± 16 (NMD) 48 ± 15 (lung diseases)
Raveling et al. [33]		Hypercapnia improvement as a survival predictor	240 COPD patients starting NIV	49.9 ± 15.0 (all patients) 54.6 ± 14.3 (stable patients) 47.9 ± 14.9 (patients after ARF)

Data are presented as mean (SD)

*NIV* noninvasive ventilation, *IV* invasive ventilation, *HRQL* health related quality of life, *HMV* home mechanical ventilation, *SRI SS* Severe Respiratory Insufficiency Questionnaire Summary Score, *COPD* chronic obstructive pulmonary disease, *TB* tuberculosis sequelae, *OHS* obesity hypoventilation syndrome, *OL* overlap syndrome, *NMD* neuromuscular disease, *RCDW* restrictive chest wall disease, *BPV-S/T* bilevel pressure ventilation -spontaneous/timed, *AVAPS* average volume assured pressure support

To our knowledge, no prospective study has examined one specific HMV protocol and its HRQL improving effects in a large, unselected case mix population.

The aim of this study was to describe the effect of initiation of protocolized, optimally conducted home mechanical ventilation with standardized follow-up in a real-life, mixed case group of chronic respiratory patients using a disease specific HRQL survey and to evaluate expected HRQL change patterns for these patients.

## Methods

### Study design and participants

The purpose of this prospective observational follow-up study was to evaluate, using the Hungarian validated version of the Severe Respiratory Insufficiency Questionnaire [21], HRQL change 6 months after the initiation of home mechanical ventilation in patients suffering from chronic respiratory failure, and identify possible factors influencing said changes. We enrolled patients diagnosed with chronic respiratory failure in need for long term mechanical ventilation, who were treated through the Semmelweis University Home Mechanical Ventilation Program from January 2014 to December 2018. Patients were referred to the Program either through elective workup for a chronic condition (elective initiation) or after recovery from a previously unknown chronic condition (acute initiation). All patients were enrolled based on work up during stable conditions and diagnosed according to available international guidelines [1–3]. Patients who were unable to complete the questionnaire were excluded. Patients who deceased before the study completion were noted as lost to follow up. Written informed consent was obtained from all patients included in the study. The study was approved by the ethics committee of Semmelweis University.

### Home mechanical ventilation initiation and follow-up

Home mechanical ventilation was initiated according to the Semmelweis University Home Mechanical Ventilation Program Guideline either after discharge from an acute hospitalization or during an elective hospital admission. Given the current reimbursement system utilized in Hungary, patients have the benefit of receiving patient tailored ventilation plans and equipment supply, including cough assisting devices, if needed. Mechanical ventilation was supplied through A40 or Trilogy 100 home mechanical ventilators (Koninklijke Philips N.V., Amsterdam) and built in, AIRcon or HC150 humidifiers (WILAmed GmbH, Kammerstein; Fishel Paykel Healthcare, Auckland) in pressure controlled, volume targeted mode through a noninvasive interface if possible or invasive interface if noninvasive ventilation was contraindicated or not feasible. Inspiratory time was aimed at 25% for patients with obstructive lung function characteristics and 30% for all other patients, then further tuned for optimal patient comfort. Supplementary O_2_ was applied if arterial blood gas values showed a p_a_O_2_ below 60 mmHg. Treatment goals for home mechanical ventilation were normalization of p_a_CO_2_ and p_a_O_2_ blood gas levels and adequate respiratory secretion management. Patients received individually tailored therapy with adequate interface (nasal, full, total face masks or cuffed tracheostomy tubes with reusable inner cannula), personalized daily ventilation plans and cough assisting device (CoughAssist T70, Koninklijke Philips N.V., Amsterdam) and/or tracheal suction devices if peak expiratory flows were below 2.5 L/s, suggesting insufficient coughing. Home mechanical ventilation was overseen by a voluntary family member or caregiver trained by our institution in skills specific to the patient’s treatment plan. Patients did not receive other institutionalized assistance. Optimal care was achieved by frequent physician follow up to achieve high compliance with therapy and continuously maintained treatment goals. Patients were followed up monthly or bimonthly by a physician of the program, trained in home mechanical ventilation. Data were not routinely collected for the purpose of this study during these physician visits.

### Data collected

Demographic data (age, sex, initial diagnosis), treatment characteristics (initiation type, interface, daily ventilation need, O_2_ supplementation need), arterial blood gas values and lung function tests were collected at baseline, ventilator settings and blood gas values (if feasible) were collected at 6 months. Arterial blood gas sampling was performed minimum 15 min after discontinuing ventilation and/or oxygen supplementation, unless patients were ventilator dependent and could not be disconnected for even short periods of time. Lung function tests were performed with the Piston PinkFlow meter (Piston Ltd., Budapest, Hungary). Initial diagnosis leading to chronic respiratory failure and long-term mechanical ventilation need was identified as chronic obstructive pulmonary disease (COPD), restrictive chest wall disease (RCWD), obesity hypoventilation syndrome (OHS), slowly progressing neuromuscular disease (NMD) or progressive neuromuscular disease (amyotrophic lateral sclerosis) (ALS).

### HRQL assessment

HRQL was assessed at baseline and 6 months after initiation of home mechanical ventilation using the Hungarian version of the SRI Questionnaire. The questionnaire consists of 49 statements and a five-point Likert scale. The results are interpreted in 7 different subscales (SRI-RC: Respiratory complaints, SRI-PF: Physical functioning, SRI-AS: Attendant symptoms and sleep, SRI-SR: Social relationships, SRI-AX: Anxiety, SRI-WB: Psychological well-being and SRI-SF: Social functioning) and the Summary Score (SRI-SS), with a value between 0 to 100, with 100 being the highest score. Patients were asked to fill out self-administered questionnaires and were assisted by caregivers if physical limitation or eyesight problems prohibited participation. The baseline questionnaire was filled out and collected during participating patients’ hospital stay, while the follow-up questionnaires were collected by the visiting physician. Patients referred to our Program after acute hospitalization were asked to base their baseline answers on the last month prior to their acute illness to reflect chronic health status.

### Statistical analysis

Continuous variables are described with means and standard deviation (±SD) and categorical variables with frequencies and percentages (n and %). SRI scores were analyzed with Paired t-test or, in case of non-normally distributed data, Signed Rank Test. Characteristics were compared with t-test or, in case of non-normally distributed data, Mann-Whitney U Rank sum test. Diagnostic groups were compared with one-way ANOVA (Brown-Forsythe) or, in case of non-normally distributed data, ANOVA on Ranks. Pearson Product Moment Correlation was used for correlation analysis. A *p*-value of < 0.05 was considered statistically significant. Analyses were conducted using SigmaPlot 12 (Systat Software, San Jose, United States) and SPSS Statistics for Windows 25 (IBM Corp., Armonk, NY). Bubble charts, commonly used to depict more complex, multidimensional correlations visually, were used to visualize quality of life change patterns in the following three dimensions: 1) size representing extent of change (larger bubbles mean larger changes), 2) texture representing direction of change (bubbles with – sign representing negative change, while bubbles with no texture representing positive change), and 3) shading representing significance (non-significant bubbles are white, while significant bubbles are gray). Bubble charts were created in Microsoft Excel.

## Results

### Patients

Out of the 75 patients enrolled, 2 were excluded because they were unable to complete HRQL forms due to cognitive impairment. One patient was deceased before study completion, and 6 patients were excluded because of missing data. A total of 66 patients completed the study.

Baseline characteristics of the patients are listed in Table 2.

**Table 2:** Baseline demographics and patient characteristics

Characteristic	mean (SD) or n (%)
Total	66 (100%)
Gender	
Female	20 (30.3%)
Male	46 (69.7%)
Age (years)	51.5 (±18.1)
Initiation of ventilation	
Acute	40 (60.6%)
Elective	24 (39.4%)
Interface	
Invasive	14 (21.2%)
Noninvasive	52 (78.8%)
O_2_ supplementation need	
No	33 (50%)
Yes	33 (50%)
O_2_ flow (L/min)	1.8 (±2.8)
Daily ventilation need (hours)	12.6 (±6.5)
Ventilator settings	
Target volume (mL)	541 (±129)
Inspiratory pressure (cmH2O)	22.2 (±4.8)
Exspiratory pressure (cmH2O)	8.3 (±3.6)
Frequency (/min)	15.9 (±3.6)
Initial diagnosis	
Chronic obstructive pulmonary disease	9 (13.6%)
Restrictive chest wall disease	5 (7.6%)
Obesity hypoventilation syndrome	20 (30.3%)
Non progressive neuromuscular disease	19 (28.8%)
Progressive neuromuscular disease (amyotrophic lateral sclerosis)	13 (19.7%)
Lung function	
FVC%	47.2 (±22.2)
FEV1%	38.7 (±21.3)
FEV1/FVC%	86.0 (±21.8)
PEF%	36.5 (±20.6)
Arterial blood gas	
p_a_O_2_ (mmHg)	69.7 (±14.2)
p_a_CO_2_ (mmHg)	49.7 (±17.5)
HCO_3_ (mmol/L)	28 (±5.2)

Data are presented as mean (SD) for continuous and as percentages (n) for categorical variables. Lung function parameters are presented as percentage of expected value

*FVC* forced vital capacity, *FEV1* forced expiratory volume in 1 s, *PEF* peak expiratory flow

Indication for invasive ventilation was bulbar symptoms in 10 (71.4%) and more than 16 h of ventilation in 4 (28.6%) of the 14 cases in the study. Invasively ventilated patients had higher daily ventilation need (17.9 (±6.5) vs. 11.1 (±5.8) hours, *p* < 0.001), lower FVC% (31.5 (±23.3) vs 51.1 (±20.3), *p* = 0.004) and PEF% (23.9 (±20.9) vs 39.6 (±19.4), *p* = 0.013) values, but had corrected blood gas parameters (pO_2_: 78.4 (±16.8) vs 67.4 (±12.7), *p* = 0.016; pCO_2_: 36.2 (±7.3) vs 53.2 (±17.7), *p* = 0.001) compared to noninvasively ventilated patients at baseline.

Patients initiated after acute hospitalization had no significant differences in baseline ventilation need, supplementary oxygen need, lung function or blood gas values compared to those enrolled electively.

Compliance remained stable during the study duration, with slightly diminishing daily ventilator use at 6 months (12.6 (±6.6) vs 11.2 (±6.6) hours, *p* < 0.001). Blood gas parameters showed improvement from baseline values (pO_2_: 69.7 (±14.2) vs 73.7 (±14.3), *p* = 0.011; pCO_2_: 49.7 (±17.5) vs 45.1 (±11.4), *p* = 0.005; HCO_3_: 28.0 (±5.2) vs 26.9 (±3.3), *p* = 0.038), despite decrease in O_2_ supplement use (1.8 (±2.8) vs 1.3 (±2.2), *p* = 0.011).

### Baseline HRQL

**Fig. 1:**
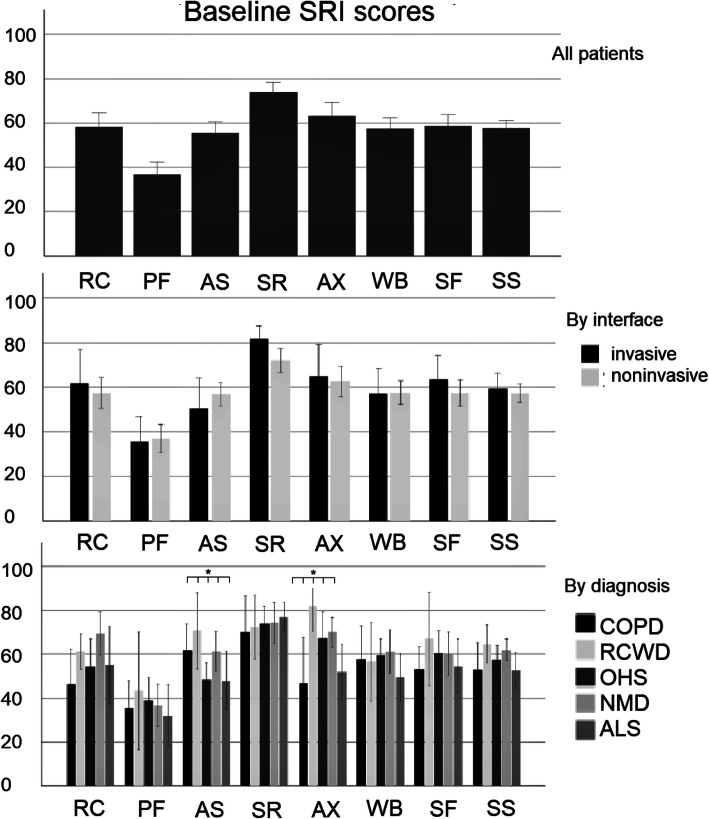
Bar graphs of baseline scores of the SRI subscales for the whole study group, by interface and by diagnosis. Boxes represent means, error bars represent standard error. Significant differences are marked with asterisk. COPD: chronic obstructive pulmonary disease, RCWD: restrictive chest wall disease, OHS: obesity hypoventilation syndrome, NMD: neuromuscular disease, ALS: amyotrophic lateral sclerosis, RC: Respiratory complaints, PF: Physical functioning, AS: Attendant symptoms and sleep, SR: Social relationships, AX: Anxiety WB: Psychological well-being, SF: Social functioning, SS: Summary Score

Overall SRI score was 57.7 (±14.4) and several subscales showed values under 60, corresponding to limited HRQL with SRI-RC, −PF, −AS, −WB and -SF being the aspects with the lowest values (Fig. 1).

#### Differences in baseline HRQL scores by interface and diagnosis

As can be seen from Fig. 1, none of the baseline SRI subscales differed in patients treated through invasive or noninvasive interface. Baseline SRI-AS and -AX subscales were significantly associated with initial diagnosis (*p* = 0.048 and *p* = 0.018 respectively). The SRI-AS scores were the lowest in OHS and ALS patients, while SRI-AX scores were the lowest in COPD and ALS patients (Fig. 1).

#### Differences in baseline HRQL scores by initiation type and O_2_ need

In addition, there was no difference in baseline SRI scores in patients initiated acutely compared to those initiated electively, or in patients needing O_2_ supplementation compared to patients who did not. Furthermore, SRI subscales showed no correlation with initial blood gas values, lung function test parameters, or hours of ventilation need (data not shown).

### Overall HRQL change at 6-month follow-up

There was an 10.5% overall improvement of SRI summary scores from baseline to six-months (57.7 ± 14.4 vs. 68.2 ± 15.8, *p* < 0.001). All SRI subscales showed significant improvement during the first 6 months of home mechanical ventilation (Table 3.)

**Table 3:** HRQL subscale values before and 6 months after initiation of home mechanical ventilation

	Quality of life before home mechanical ventilation	Quality of life 6 months after initiation of home mechanical ventilation	
RC	58.3 (±25.9)	78.7(±17.3)	*p* = 0.015*
PF	36.7(±22.5)	42.8(±29.6)	*p* < 0.001*
AS	55.5(±20.6)	74.8 (±14.0)	*p* = 0.006*
SR	74.1(±18.1)	76.2(±18.4)	*p* < 0.001*
AX	63.3(±25.0)	77.7(±22.6)	*p* < 0.001*
WB	57.5(±19.7)	63.8(±19.3)	*p* < 0.001*
SF	58.7(±21.1)	63.6(±24.6)	*p* < 0.001*
SS	57.7(±14.4)	68.2(±15.8)	*p* < 0.001*

Significant differences are marked with asterisk

*RC* Respiratory complaints, *PF* Physical functioning, *AS* Attendant symptoms and sleep, *SR* Social relationships, *AX* Anxiety, *WB* Psychological well-being, *SF* Social functioning, *SS* Summary Score

### Factors effecting HRQL change

#### HRQL change by interface and diagnosis

**Fig. 2:**
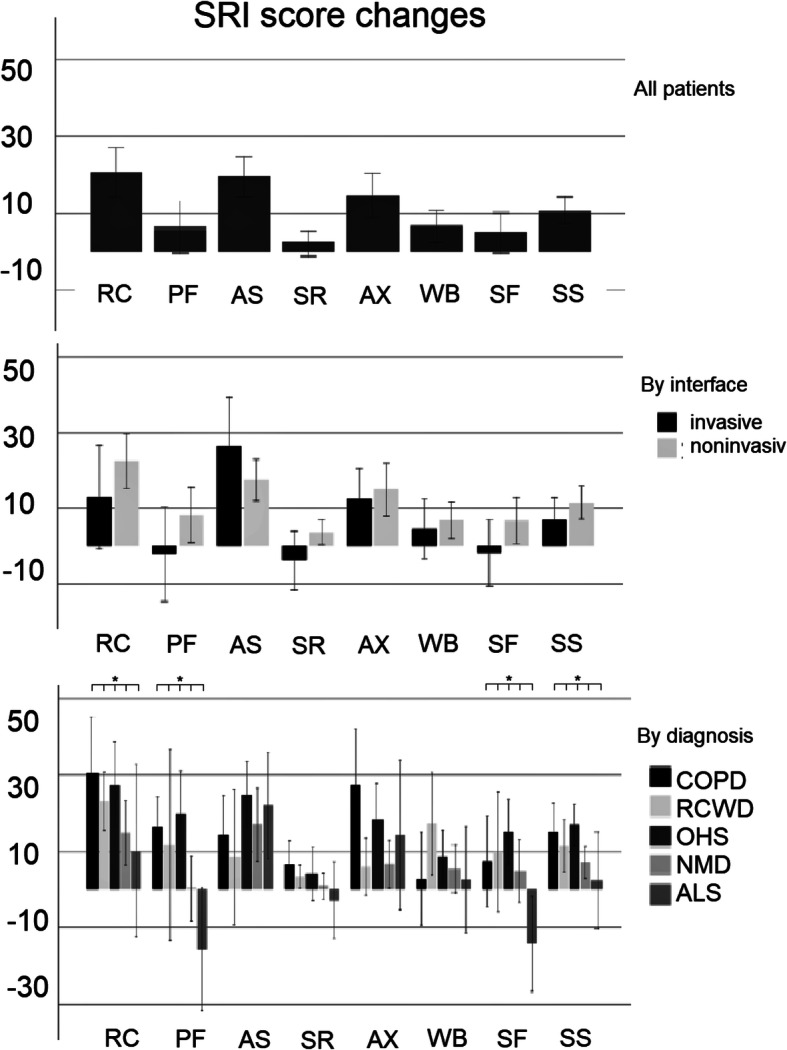
Bar graphs of changes in the scores of SRI subscales at the 6-month follow-up in the whole study group, by interface and by diagnostic groups. Boxes represent means, error bars represent standard error. Significant differences are marked with asterisk. COPD: chronic obstructive pulmonary disease, RCWD: restrictive chest wall disease, OHS: obesity hypoventilation syndrome, NMD: neuromuscular disease, ALS: amyotrophic lateral sclerosis, RC: Respiratory complaints, PF: Physical functioning, AS: Attendant symptoms and sleep, SR: Social relationships, AX: Anxiety WB: Psychological well-being, SF: Social functioning, SS: Summary Score

As can be seen from Fig. 2, the choice of interface did not affect change in SRI subscales (*p* = 0.660). Changes in SRI-RC, −PF, −SF and -SS subscales were significantly influenced by initial diagnosis (*p* = 0.025, *p* < 0.001, *p* = 0.002 and *p* = 0.025 respectively).

**Fig. 3:**
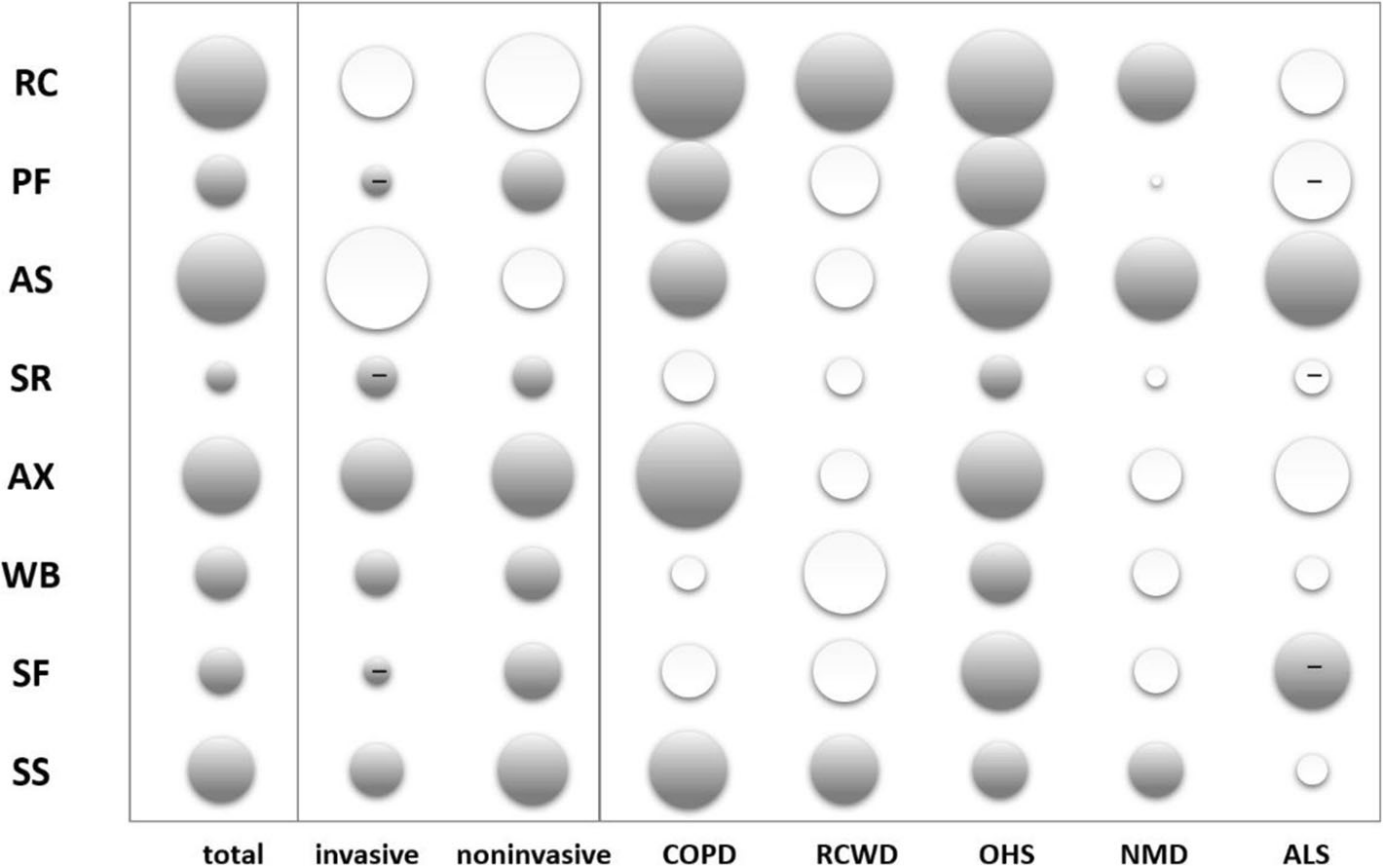
Bubble chart of SRI subscale changes for the whole study group and according to interface and diagnosis after 6 months of home mechanical ventilation. Size corresponds to value of change from baseline. Significant changes are marked with gray shading. Negative changes are marked by negative sign pattern. COPD: chronic obstructive pulmonary disease, RCWD: restrictive chest wall disease, OHS: obesity hypoventilation syndrome, NMD: neuromuscular disease, ALS: amyotrophic lateral sclerosis, RC: Respiratory complaints, PF: Physical functioning, AS: Attendant symptoms and sleep, SR: Social relationships, AX: Anxiety WB: Psychological well-being, SF: Social functioning, SS: Summary Score

When further analyzing HRQL changes within diagnostic groups, we found that different diagnostic groups had different HRQL change patterns, which is visualized in the bubble chart depicting relative changes in different patient groups (Fig. 3). As can be seen from the figure, the patients benefiting most from HMV were COPD patients, while OHS patients improved across the most SRI subscales. SRI-RC subscale improved in all groups but ALS patients, and most prominently in COPD and OHS patients. SRI-PF and -AX scores improved only in COPD and OHS patients. SRI-SR subscales showed no change in any of the patient groups, except for OHS patients. SRI-WB and-SF improved only in OHS, and SRI-SF actually declined significantly in ALS patients. Overall SRI-SS scores improved in all patient groups except for ALS (see also Additional file 1).

#### HRQL change by initiation type, O_2_ need, and baseline HRQL

Summary score improved significantly more in patients initiated acutely compared to patients initiated electively (12.3 ± 16.8 vs. 7.4 ± 9.4, *p* = 0.029). Change in SRI subscales showed no correlation with initial blood gas values, lung function test parameters or hours of ventilation need (data not shown). In patients using O_2_ supplementation SRI-PF, −SF and -SS subscales improved significantly compared to patients that did not require O_2_ supplementation (SRI-PF: 13.1 ± 25.5 vs. -1.0 ± 25.0 *p* = 0.001; SRI-SF: 9.2 ± 21.9 vs.0.6 ± 20.3, *p* = 0.022; SRI-SS: 12.0 ± 15.6 vs. 9.0 ± 13.8, *p* = 0.006).

**Fig. 4:**
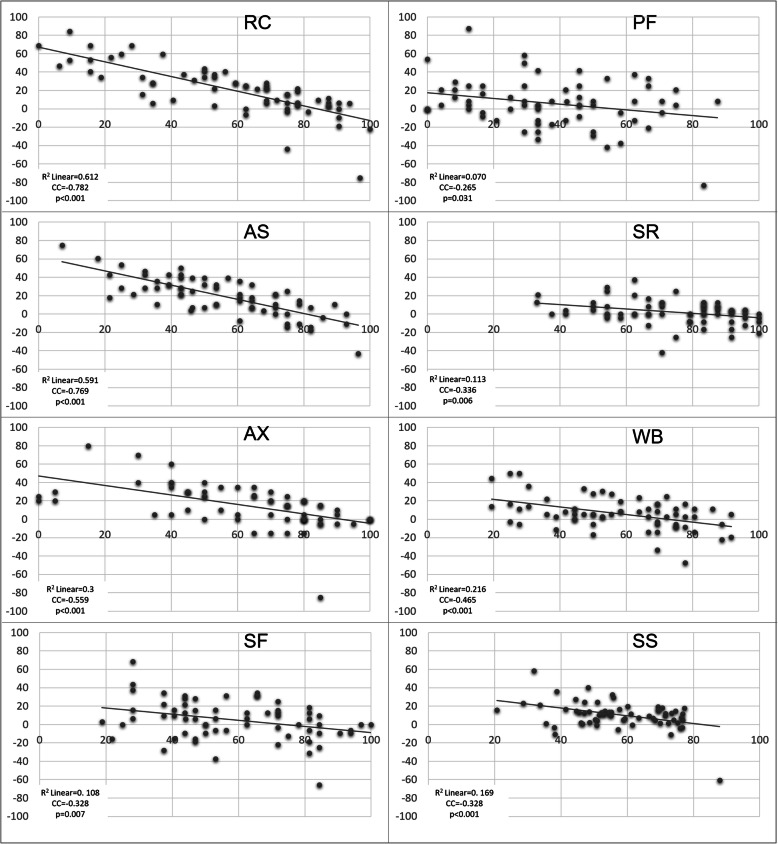
Subscale changes in relation to baseline subscales. Scatterplot of SRI subscale changes according to initial SRI values. X axis shows change of SRI subscale. Y axis shows initial SRI subscale value. R^2^Linear: coefficient of determination, CC: correlation coefficient, RC: Respiratory complaints, PF: Physical functioning, AS: Attendant symptoms and sleep, SR: Social relationships, AX: Anxiety WB: Psychological well-being, SF: Social functioning, SS: Summary Score

When further analyzing factors affecting HRQL changes in patients receiving home mechanical ventilation, we found that change in SRI subscales showed significant correlation with baseline SRI scores (SRI-RC: *p* < 0.001; CC = -0.782; SRI-PF: *p* = 0.0314; CC = -0.265; SRI-AS *p* < 0.001; CC = -0.769; SRI-SR: *p* = 0.006; CC = -0.336; SRI-AX: *p* < 0.001; CC = -0.559; SRI-WB: *p* < 0.001; CC = -0.465; SRI-SF: *p* = 0.007; CC = -0.328; SRI-SS: *p* < 0.001; CC = -0.411) (Fig. 4). This phenomenon was most prominent in SRI-RC and SRI-AS scales.

## Discussion

The aim of our study was to assess HRQL change induced in a mixed case chronic respiratory failure patient population in one HMV center. Our cohort included patients with a variety of underlying conditions, ventilated both invasively and noninvasively, initiated acutely and electively, resulting in a real-life study population. Frequent follow up visits ensured optimal compliance and maintained treatment goals, ensuring that HRQL changes were comparable despite the mixed case study population.

We found that overall HRQL improved significantly in the first 6 months of home mechanical ventilation and the grade of HRQL change was influenced by initial diagnosis. Furthermore, we also found that patients with worse initial HRQL, initiated acutely and needing O_2_ supplementation, had a greater improvement in HRQL and that HRQL did not improve in ALS patients but was maintained despite significant progression of the disease.

The group of patients recruited for this study had baseline characteristics similar to home mechanical ventilation populations described before (see Tables 1 and 2). Noninvasively ventilated patient populations have been known to be characterized by a daily ventilation need of 6.5 to 9 h, FVC% values of 42–62%, pO_2_ of 66–73 Hgmm, and pCO_2_ of 44–59, while invasively ventilated patients have been reported to have higher daily ventilation needs (18–20 h), with O_2_ supplementation needed in 75% of patients [12–14, 22]. In our study population, invasive and noninvasive ventilation ratio was similar to proportions noted in the Eurovent study describing patterns of HMV in Europe [5]. This ratio explains the mean daily ventilator use in our study as well as the high O_2_ supplementation need. Lung function tests and blood gas values at baseline were consistent with values described in the previous references, with mean values showing restrictive lung function changes, hypoxia and hypercapnia [12–14, 22]. The majority of patients were initiated on HMV after resolution of an acute exacerbation of a previously undiagnosed chronic condition, which might be explained by possible insufficient diagnostic algorithms and screening of patient populations with higher risk of chronic respiratory failure.

Baseline HRQL in our study population was similar (SRI-SS: 57.7 ± 14.4) to values described in patients suffering from chronic respiratory failure (see Table 1), with the subscales of Respiratory complaints, Attendant symptoms and sleep, Physical functioning, Social functioning and Psychological well-being being the aspects most diminished. To our knowledge, no previous study compared patients ventilated invasively versus patients ventilated noninvasively, although patients ventilated through a tracheostomy are generally assumed to have diminished HRQL [14, 34]. In our study population none of the baseline SRI subscales differed in patients treated through invasive or noninvasive interface, in patients initiated acutely or electively or in patients needing O_2_ supplementation compared to patients who did not, and SRI subscales showed no correlation with initial blood gas values, lung function test parameters or hours of ventilation need, pointing to the fact that HRQL is not merely influenced by the severity of respiratory failure, although the relatively low number of invasively ventilated patients might contribute to this finding.

We found that quality of life increased significantly during the six-month observation period (SRI summary score changed from 57.7 ± 14.4 vs. 68.2 ± 15.8, *p* < 0.001), which is similar to changes reported by studies in more selective patient populations (see Table 1). HRQL subscales that were most robustly improved were Respiratory complaints and Attendant symptoms and sleep, which are indeed the main goals of long-term mechanical ventilation. Anxiety subscales also showed significant improvement, which is most likely a consequence of the improvement of the two prior scales. Social relations and Social functioning showed no change, which suggests that these subscales are influenced by more complex disease attributes and are not solely related to respiratory symptoms. The change in Physical functioning also showed no significant overall improvement, which is understandable since several patient groups had stable or progressive neuromuscular impairment. When looking at specific disease groups, we found that diseases where neuromuscular involvement was not present (COPD and OHS), Physical functioning subscale actually improved significantly and considerably (16.2 ± 12.2, *p* = 0.002 and 19.8 ± 24.9, *p* < 0.001 respectively).

The overall SRI HRQL change found in our study is similar to changes previously reported in a study using solely noninvasive ventilation [13]. Furthermore, our study found higher post-ventilation HRQL values than a study in a population ventilated invasively [14]. As an additional interesting finding, our study suggests that an increase in HRQL does not depend on interface [35], meaning HRQL can both be increased through invasive and noninvasive ventilation. This is important because there is a general fear towards tracheostomy in patients requiring long term mechanical ventilation [36]. The results of our study provide evidence that significant improvement might be achieved in HRQL even through an invasive interface. This can be attributed to patient-tailored home mechanical ventilation therapy, which is feasible under current reimbursement system in Hungary and permits complex ventilation and physiotherapy plans for patients, despite vastly different needs. The other possible reason for the consistently improved outcome might be the frequent follow-ups provided by our program. Current international guidelines do not specify the frequency of follow up for home mechanically ventilated patients [1–3], but most centers reduce the frequency of home or ambulatory visits to twice a year after successful initiation [4, 7]. In our patient population, however, follow-up was provided monthly for patients ventilated invasively and monthly or bi-monthly for stable patients ventilated through a noninvasive interface.

When looking at other factors potentially influencing HRQL change, we found that HRQL increase was the greatest among patients with lower initial HRQL scores, patients initiated with long term mechanical ventilation in an acute setting, and patients needing supplemental O_2_ therapy, implying that a lot of patients included in the current study might have received home mechanical ventilation relatively late in their course of disease progression. This is corroborated by the study previously published by our group that describes the current characteristics and prevalence of HMV in Hungary [37], which found that prevalence of home mechanical ventilation is lower than that found in other internationally published data, because many ventilator-dependent patients might not receive therapy in time. This finding is especially important, since there is still no clear data to help us differentiate between long term therapeutic home mechanical ventilation and palliative care with ventilation. The results of our study suggest that even patients with very poor HRQL benefit greatly from initiation of home mechanical ventilation.

Our results regarding the HRQL influencing effect of initial diagnosis corroborate the findings published previously [13, 14]. Based on our findings, COPD patients can expect the most benefit in HRQL increase, which is important, since long term home mechanical ventilation is still controversial in this indication [38–40], although it should be noted that COPD is a heterogenous disease group and our study did not analyze disease phenotypes. Moreover, when analyzing patient groups, we found that OHS patients improved most consistently, *on par* with recently published guidelines [41]. Given that this disease effects a younger population and its main symptoms are debilitating daytime sleepiness and hypercapnia [42], this HRQL improvement has important socioeconomical consequences. It is also important to note that recent guidelines indicate most OHS patients might be managed with initial CPAP therapy. Prospective HRQL change studies might verify whether optimal improvement can be achieved by this approach.

In our study, the only patient group not showing improved HRQL scores were the ALS patients, which is *on par* with literature describing this progressive neuromuscular disease [43], although noninvasive ventilation has been shown to improve HRQL and even survival for a shorter period of time [44]. In our study, spanning a longer period, it is especially conspicuous that ALS patients were the only ones to show significant detriment in one subscale (SRI Social functioning change), suggesting that these patients often become isolated during the progression of their disease. However, it is noteworthy that despite HRQL subscale changes due to disease progression, overall Summary scores remained stable, mainly because Attendant symptoms and sleep, Anxiety and Respiratory complaints subscales counterbalance the decrease in Social functioning and Physical functioning subscales. This suggests that home mechanical ventilation might be a valid palliative care technique in ALS even in cases where significant lifespan or HRQL improvement can’t be proven.

The main limitation of our study is the sample size that might be considered small; however previously published studies on HMV patients utilized similar sample sizes [11, 13, 14, 21, 23]. Our study was a single center, prospective HRQL change investigation under optimized care using the SRI Questionnaire, providing valuable “real-life” information on a mixed case population previously not extensively described in the literature. Another limitation is that the study was not designed to identify outcome differences in patients with different interfaces or initial diagnosis, nor was powered for multivariate analysis to exactly define confounding factors influencing subscale changes, as subgroup analysis would have resulted in groups that would have been too small to deduct relevant clinical correlations.

## Conclusions

Starting home mechanical ventilation is accompanied by improved quality of life in several patient groups suffering from chronic respiratory failure. Our findings suggest that HRQL improvement is independent of classic markers of the severity of chronic respiratory failure (e.g., baseline lung function, arterial blood gas value, hours of ventilation need) or interface used for ventilation, but it is dependent on the type of disease causing the chronic respiratory failure, initiation type (acute versus elective), initial HRQL and O_2_ supplementation need. Our results further suggest that acutely initiated, O_2_ dependent COPD and OHS patients with low initial HRQL can expect the most benefit, while ALS patients can expect maintenance of overall HRQL despite ongoing neurological deterioration. Further prospective studies are needed to confirm our findings and to identify whether HRQL changes correlate with disease progression and mortality, and how often HRQL needs to be measured during the follow up of patients receiving home mechanical ventilation in order to better improve the lives of patients suffering from chronic respiratory failure.

## Supplementary information

**Additional file 1.** HRQL subscale changes according to diagnostic groups. Contains exact numerical HRQL subscale changes according to diagnostic groups with significant values noted.

## Data Availability

The datasets used and analyzed during the current study are available from the corresponding author on reasonable request.

## Abbreviations

ALS: Amyotrophic lateral sclerosis
ANOVA: Analysis of variance
AVAPS: Average volume assured pressure support
BPV-S/T: Bilevel pressure ventilation -spontaneous/timed
CC: Correlation coefficient
COPD: Chronic obstructive pulmonary disease
FEV1: Forced expiratory volume in 1 s
FVC: Forced vital capacity
HMV: Home mechanical ventilation
HRQL: Health related quality of life
NMD: Neuromuscular disease
OHS: Obesity hypoventilation syndrome
p_a_CO_2_: Arterial partial pressure of carbon dioxide
p_a_O_2_: Arterial partial pressure of oxygen
PEF: Peak expiratory flow
RCWD: Restrictive chest wall disease
SD: Standard deviation
SRIQ: Severe Respiratory Insufficiency Questionnaire
SRI-AS: Attendant symptoms and sleep
SRI-AX: Anxiety
SRI-PF: Physical functioning
SRI-RC: Respiratory complaints
SRI-SF: Social functioning
SRI-SR: Social relationships
SRI-SS: Summary Score
SRI-WB: Psychological well-being
