# Supplementary material for: Home mechanical ventilation: quality of life patterns after six months of treatment
Source: BMC Pulm Med. 2020 Aug 17;20:221. doi: 10.1186/s12890-020-01262-z (PMC7433042; doi:10.1186/s12890-020-01262-z)
Supplement: Supplementary file 1 — Additional file 1. HRQL subscale changes according to diagnostic groups. Contains exact numerical HRQL subscale changes according to diagnostic groups with significant values noted. [file 12890_2020_1262_MOESM1_ESM.docx]

QoL subscale changes according to diagnostic groups.

| SRI subscale change | COPD (9) | RCWD (5) | OHS (20) | NMD (19) | ALS (13) |
| --- | --- | --- | --- | --- | --- |
| Respiratory complaints | 30.6±21.8  p=0.003* | 23.1±8.5  p=0.004* | 27.3±25.1  p<0.001* | 14.8±18.4  p<0.001* | 10.1±40.6  p=0.387 |
| Physical functioning | 16.2±12.2  p=0.002* | 11.7±28.0  p=0.405 | 19.8±24.9  p<0.001* | 0.2±18.5  p=0.963 | -15.7±28.9  p=0.074 |
| Attendant symptoms and sleep | 14.3±15.5  p=0.012* | 8.6±20.0  p=0.392 | 24.8±19.5  p<0.001* | 16.9±20.9  p=0.002* | 22.0±25.2  p=0.008* |
| Social relationships | 6.5±9.6  p=0.077 | 3.3±3.5  p=0.099 | 4.2±15.5  p=0.243 | 0.9±7.6  p=0.617 | -2.9±18.0  p=0.579 |
| Anxiety | 27.3±22.1  p=0.006* | 6.0±8.2  p=0.178 | 18.3±21.2  p=0.001* | 6.6±13.8  p=0.053 | 14.2±35.2  p=0.052 |
| Psychological well-being | 2.8±18.3  p=0.664 | 17.2±15.0  p=0.062 | 8.6±15.6  p=0.023* | 5.4±13.5  p=0.099 | 2.6±24.8  p=0.716 |
| Social functioning | 7.3±17.8  p=0.254 | 10.0±17.6  p=0.271 | 15.0±19.0  p=0.002* | 4.9±18.1  p=0.25 | -14.2±22.7  p=0.043* |
| Summary Score | 15.0±11.6  p=0.005* | 11.4±7.6  p=0.029* | 7.5±43.6  p<0.001* | 7.1±9.1  p=0.003* | 2.3±22.9  p=0.376 |

Data are presented as mean (±standard deviation). Significant values are marked with bold font and asterix. Patient numbers within groups are marked as (n). Subscale showing significant reduction is marked with a minus sign. COPD: chronic obstructive pulmonary disease, RCWD: restrictive chest wall disease, OHS: obesity hypoventilation syndrome, NMD: slowly progressing neuromuscular disease, ALS: amyotrophic lateral sclerosis
